# Endothelial Relaxation Mechanisms and Oxidative Stress Are Restored by Atorvastatin Therapy in Ovariectomized Rats

**DOI:** 10.1371/journal.pone.0080892

**Published:** 2013-11-21

**Authors:** Izabela Facco Caliman, Aline Zandonadi Lamas, Polyana Lima Meireles Dalpiaz, Ana Raquel Santos Medeiros, Glaucia Rodrigues Abreu, Suely Gomes Figueiredo, Lara Nascimento Gusmão, Tadeu Uggere Andrade, Nazaré Souza Bissoli

**Affiliations:** 1 Department of Physiological Sciences, Federal University of Espirito Santo, Vitória, Espirito Santo, Brazil; 2 Biological and Health Sciences, Federal Institute of Espirito Santo, Vitória, Espirito Santo, Brazil; 3 Department of Pharmacy, University Center of Vila Velha, Vila Velha, Espirito Santo, Brazil; VCU, United States of America

## Abstract

The studies on hormone replacement therapy (HRT) in females with estrogen deficiency are not conclusive. Thus, non-estrogen therapies, such as atorvastatin (ATO), could be new strategies to substitute or complement HRT. This study evaluated the effects of ATO on mesenteric vascular bed (MVB) function from ovariectomized (OVX) female rats. Female rats were divided into control SHAM, OVX, and OVX treated with 17β-estradiol (EST) or ATO groups. The MVB reactivity was determined in organ chambers, vascular oxidative stress by dihydroethidine staining, and the expression of target proteins by western blot. The reduction in acetylcholine-induced relaxation in OVX rats was restored by ATO or EST treatment. The endothelium-dependent nitric oxide (NO) component was reduced in OVX rats, whereas the endothelium-derived hyperpolarizing factor (EDHF) component or prostanoids were not altered in the MVBs. Endothelial dysfunction in OVX rats was associated with oxidative stress, an up-regulation of iNOS and NADPH oxidase expression and a down-regulation of eNOS expression. Treatment with ATO or EST improved the NO component of the relaxation and normalized oxidative stress and the expression of those signaling pathways enzymes. Thus, the protective effect of ATO on endothelial dysfunction caused by estrogen deficiency highlights a significant therapeutic benefit for statins independent of its effects on cholesterol, thus providing evidence that non-estrogen therapy could be used for cardiovascular benefit in an estrogen-deficient state, such as menopause.

## Introduction

The role of estrogens in vascular function has received considerable research interest because epidemiological studies have shown a greater risk of developing cardiovascular disease (CVD) due to reduced 17β-estradiol levels after menopause [1–3]. One of the interesting factors is the proposed interaction between estrogens and endothelial factors [4,5]. 

The main mechanisms involved in the impaired vascular response in estrogen deficiency models are connected decreased nitric oxide (NO) bioavailability and the attenuation of hyperpolarization and relaxation transduced by endothelium-derived hyperpolarizing factor (EDHF) [6–8]. This impaired vascular response may occur in long-term (ovariectomy) and short-term (diestrous cycle) estrogen-deficient states [6]. In addition, impaired endothelial function in ovariectomized rats was associated with an increase in superoxide anion production and the increased protein expression of NADPH oxidase subunits, as gp91phox and p22phox [9,10].

Recent experimental and clinical evidence has suggested that statins (i.e., 3-hydroxy-3-methylglutaryl coenzyme A (HMG-CoA) reductase inhibitors) have cholesterol-independent (“pleiotropic”) effects. Statins are extremely safe but can produce myalgia and rarely rhabdomyolysis [11]. Additionally, the risk of the development of diabetes in patients with impaired fasting glucose, metabolic syndrome or severe obesity was reported by some statin therapy studies [12,13]. However, these side effects do not exceed the benefits promoted by the hypercholesterolemia therapy [14,15]. More importantly, even postmenopausal patients show a significant reduction of atherosclerosis after being treated with statins [16,17]. 

Like estrogen, statins exert vasoprotective effects that are independent of their lipid-lowering action [18–20]. The results from human and animals studies have helped to understand the mechanisms of action for statins in the cardiovascular system and have relevant clinical implications [20–24] related to variations in the lipid profile [25] and the effect on the vessel wall [26,27]. Statins can improve endothelial function through attenuating vascular and myocardial remodeling and by inhibiting oxidation in vascular tissue and anti-inflammatory mechanisms [14,27–29]. In ovariectomized rats with endothelial dysfunction and atherosclerotic process, a combined treatment with statins and raloxifene, a selective estrogen receptor modulator, might play a potential preventive role in the early stages of atherosclerosis development decreasing the levels of inﬂammatory markers [30]. These actions reinforce the concept that a significant part of the cardiovascular actions of these drugs is exerted at the vascular level [31]. 

Although statins are able to reduce the risk of coronary events and mortality in patients with coronary artery disease [14,19], studying the action of these drugs on endothelial function in models of estrogen deficiency is necessary. Despite previous reports, there are limited data comparing the effects of statins and estrogen on the cardiovascular system, and no studies have addressed the actions of statins on vascular responses to acetylcholine (ACh) in resistance vessels. From a theoretical point of view, if statins could improve endothelial dysfunction similar to estrogen, atorvastatin therapy should improve the vascular dysfunction observed in an animal model of estrogen deficiency. Regarding to this statin, recent studies demonstrate the benefit of low- dose atorvastatin in the prevention of cardiovascular disease in the absence of dyslipidemia [32,33]. Moreover, the longer half-life of atorvastatin could contribute to a higher efficacy in reduction of cholesterol levels [34].

Therefore, we performed this study to evaluate the effects of atorvastatin on vascular reactivity in mesenteric beds from ovariectomized female rats and the involvement of NO, EDHF and NADPH oxidase in these mesenteric resistance arteries.

## Material and Methods

### Ethics Statement

All of the procedures were conducted in accordance with the biomedical research guidelines for the care and use of laboratory animals, as stated by the Brazilian College of Animal Experimentation (COBEA). The experimental protocol was approved by the Ethics Committee in Animal Experimentation of the Federal University of Espirito Santo under the number 069/2011.

### Animals

The experiments were performed using eight weeks-old female Wistar rats weighting 180 to 200 g. Throughout the experiment, the animals were housed in groups in a temperature- (22 °C) and humidity- (50%) controlled room with a 12-h (light) – 12-h (dark) cycle. Standard rat chow and tap water were available *ad libitum*. Four groups were studied (N = 6 animals per group): sham-operated females (SHAM); ovariectomized females (OVX); ovariectomized females treated with 17β-estradiol (EST: 0.5 µg/kg/day; Sigma Chemical Co., St. Louis, MO, USA) or ovariectomized females treated with atorvastatin (ATO: 20 mg/kg/day). 

### Ovariectomy and Treatments

Bilateral ovariectomy was performed in female rats under ketamine (70 mg/kg) and xylazine (10 mg/kg) anesthesia by intraperitoneal injection (i.p). The females were subjected to a muscular incision to open the peritoneal cavity for posterior connection of the uterine tubules and removal of the ovaries. Then, the peritoneal cavity was sutured and cleaned. The female sham group only underwent an incision. Next, the animals were allowed to recover. Twenty-one days after surgery, the ovariectomized female rats were subcutaneously given 17β-estradiol diluted in peanut oil (EST group) or atorvastatin through gavage (ATO group). The sham and ovariectomized (OVX) groups received only the vehicle. Those treatments lasted 14 days. As previously described [35], the effects of ovariectomy and estrogen treatment were confirmed by measuring the body and uterine weights at the time of the experiment.

### Estrous cycle phase determination

Daily vaginal smears were taken from each female sham rat as previously described [36] to confirm that their estrous cycles were proceeding normally [(i) estrus, (ii) metaestrus, (iii) diestrus, and (iv) proestrus]. The vaginal epithelial cells were examined via a microscope for at least 7 consecutive days before the experiment. The swabs were performed between 8:00 and 10:00 A.M. to maintain consistency. The females with a normal estrous cycle were killed during the proestrus phase.

### Cholesterol measurement

Blood samples were collected on the day of the vascular reactivity experiments by aortic puncture, after the cannulation of the superior mesenteric artery. All of the samples were centrifuged (1500 *g* for 10 minutes), and serum was stored at −20 °C. The LDL- and total- serum cholesterol were measured using automated equipment (COBAS 6000 Analyzer, Roche Diagnostics, SA). 

### Vascular reactivity in the mesenteric vascular bed

After the treatment period, the rat mesenteric vascular bed (MVB) was isolated according to McGregor [37]. Briefly, the rats were anesthetized with ketamine and xylazine (70 and 10 mg/kg, i.p., respectively) and the superior mesenteric artery, with its branches, was isolated and perfused *in vitro* with oxygenated (95% O_2_- 5% CO_2_) Krebs-Henseleit solution (130 mM NaCl, 4.7 mM KCl, 1.6 mM CaCl_2_.2H_2_O, 1.17 mM MgSO_4_.6H_2_O, 1.18 mM NaHCO_3_, 1.6 mM KH_2_PO_4_, 14.9 mM EDTA, and 11.1 mM glucose, pH 7.4) at a constant flow rate of 4 ml/min and maintained at 37 °C. The MVB were excised from the intestinal wall, placed in a chamber, and the preparations were allowed to stabilize for 30 min before the beginning of the experiments. Changes in the perfusion pressure, which reflect peripheral resistance, were measured with a pressure transducer, (Spectramed P23XL) connected to an acquisition system (MP100A, BIOPAC System, Inc., Santa Barbara, USA), and were calculated as percentage of reduction in the perfusion pressure after noradrenaline (NE)-induced contraction. After a stabilization period, noradrenaline (0.1 to 0.3 mM) was added to the perfusion fluid to increase the tone by approximately 90-120 mmHg. Once a stable tone was established, concentration-response curves to acetylcholine (ACh; 1.68 x 10^-12^ to 1.68 x 10^-3^ M) were determined in the MVB. The ACh curves were performed initially in each MVB without any inhibitors. To evaluate the effect of NO availability on vascular reactivity, the preparations were treated with the nonspecific NOS inhibitor N^G^-nitro-L-arginine methyl ester (L-NAME, 100 mM) and the inducible NO synthase (iNOS) inhibitor aminoguanidine (AG,100 mM) [8]. The participation of EDHF in modulating endothelial function was assessed by constructing concentration-response curves to ACh in presence of L-NAME plus the cyclooxygenase (COX) inhibitor indomethacin (INDO, 2.8 µM) [38], to exclude the involvement of prostanoids and NO. All of these drugs were added to the bath 30 min before performing the ACh concentration-response curves.

### Western blot analysis

Mesenteric arteries were carefully dissected free of surrounding adipose tissue for a full MVB representation. The samples were homogenized and centrifuged at 3000 *g* for 15 minutes (4°C). Protein concentrations were determined using the method of Lowry [39–43]. The protein lysates [50 μg for eNOS, iNOS, NADPH oxidase (gp91phox) and 80 μg for COX-2], were separated by 7.5% and 10% SDS-PAGE, respectively. The proteins were transferred to polyvinylidene diﬂuoride (PVDF) membranes that were incubated with mouse monoclonal antibodies for endothelial nitric oxide synthase (eNOS, 1:2500, BD Transduction Laboratories, Lexington, KY, USA), inducible nitric oxide synthase (iNOS, 1:2000, BD Transduction Laboratories, Lexington, KY, USA), NADPH oxidase (gp91phox, 1:2000, BD Transduction Laboratories, Lexington, KY, USA) or β-actin (1:1500, Santa Cruz Biotechnology, Inc, Santa Cruz, CA, USA) or rabbit polyclonal antibody for cyclooxygenase 2 (COX-2, 1:200, Santa Cruz Biotechnology, Inc, Santa Cruz, CA, USA). After washing, the membranes were incubated with alkaline phosphatase conjugated anti-mouse IgG (1:3000, Abcan Inc, MA, USA), except for COX-2, which the incubation was with anti-rabbit IgG antibody (1:7000, Santa Cruz Biotechnology, Inc, CA, USA). The bands were visualized using an NBT/BCIP system (Invitrogen Corporation, CA, USA) and quantiﬁed using the *ImageJ* software. The results were calculated by the ratio of the density of speciﬁc bands to the corresponding β-actin. The data are from six independent experiments and are expressed as the percentage of arbitrary units in relation to the SHAM group. 

### Determination of vascular ROS formation

The redox-sensitive fluorescent dye dihydroethidine (DHE) was used to evaluate the *in situ* formation of reactive oxygen species (ROS), following a previously described method [44]. Mesenteric arterial rings (3 to 4 mm in length) were embedded in the OCT compound (Tissue-Tek) and frozen at -80°C. Transverse sections (8 µm) obtained using a cryostat were incubated at 37 °C for 30 min with phosphate buffer. Fresh phosphate buffer containing hydroethidine (2 µM) was topically applied to each tissue section, and the slices were incubated in a light-protected humidified chamber at 37 °C for 30 min to determine *in situ* ROS formation. The negative control sections received the same volume of phosphate buffer without hydroethidine. Images were obtained using an optical microscope (DM 2500, Leica Microsystems, Germany) equipped with a camera using a 40x objective. Fluorescence intensity in each section was expressed as the percentage of arbitrary fluorescence units (AFU) in relation to the SHAM group. Quantification of the staining was performed using the Image ProPlus v.6 software.

### Statistical analysis

The data are reported as the means ± S.E.M. The Prism 5 software (Graph Pad Software, San Diego, CA, USA) was used for the statistical analysis. For analyses percentage of reduction in the perfusion pressure responses in the MVB, basal pressure perfusion (P1) was elevated (P2) by addition of NE in the perfusion fluid. Thereby, in each dose of ACh, this elevated pressure was momentarily reduced (P3) in certain degree. Thus, the reduction in the perfusion pressure (%) was calculated by the reduction in the perfusion pressure induced by ACh divided by the elevation evocated by NE, according to the formula: reduction in the perfusion pressure (%) = 100[(P2-P3)/(P2-P1)]. For each concentration-response curve, the maximal response (E_max_) and -log concentration of the drug required to produce 50% of the maximum response (pD_2_) were calculated using non-linear regression analysis. To compare the effects of some drugs on the vasodilatory responses to ACh, some of the results were expressed as differences in the area under the concentration-response curves (dAUC) for the control and experimental groups. These values indicate whether the magnitude of the effect differed among the groups. For protein expression, the data were expressed as the ratio between signals on the blot corresponding to the protein of interest and β-actin. The differences were analyzed using one or two-way ANOVA followed by a Tukey test. A difference of P<0.05 was considered to be statistically significant.

## Results

### The effects of ovariectomy and treatments on body weight, uterine weight and serum cholesterol

Table 1 shows the body weight (BW), uterine weight and ratio of uterine weight to tibia length in the SHAM, OVX, EST and ATO groups. Ovariectomy led to a higher BW compared to the SHAM group. Treatment with 17β-estradiol reversed the ovariectomy-induced increase in BW (*P*<0.05); however, atorvastatin did not alter the BW in the OVX group. Conversely, ovariectomy produced a significant decrease in the uterine weight and the ratio of uterine weight to tibia length compared to the SHAM animals. ATO did not modify this parameter. The uterine weight to tibia length ratio returned to normal values after 17β-estradiol treatment. Ovariectomy neither the treatments used in the present study were able to alter the LDL- and total- serum cholesterol (Table 1). 

**Table 1:** Effect of ovariectomy and/or treatments on body weight, uterine weight and cholesterol levels.

	**Groups (n=12 per group)**			
	**SHAM**	**OVX**	**EST**	**ATO**
**Initial body weight (g)**	198.8±3.3	199.6±4.2	203.9±2.5	196.9±4.4
**Final body weight (g)**	233.4±3.7	268.4±7.3**	244.0±5.0	258.6±6.2*
**Uterine weight (g)**	0.51±0.03	0.09±0.01**	0.44±0.02	0.10±0.01**
**Uterine weight/tibia length (mg/cm)**	138.3±14.2	24.6±0.8**	114.1±4.8	26.5±1.6**
**Cholesterol (mg/dL)**	73.4±3.6	75.66±5.9	81.31±3.4	76.88±6.5
**LDL (mg/dL)**	14.20±3.46	14.94±1.59	22.67±2.91	18.61±2.87

The results are the means ± S.E.M. Parameters measured in the control SHAM, ovariectomized (OVX) and ovariectomized treated with 17β-estradiol (EST) or Atorvastatin (ATO) group. Statistical significance is indicated by **p<0.01 and *p<0.05 *vs.* the SHAM group (one-way ANOVA followed by Tukey’s test).

### Effect of atorvastatin treatment on vascular reactivity

**Figure 1:**
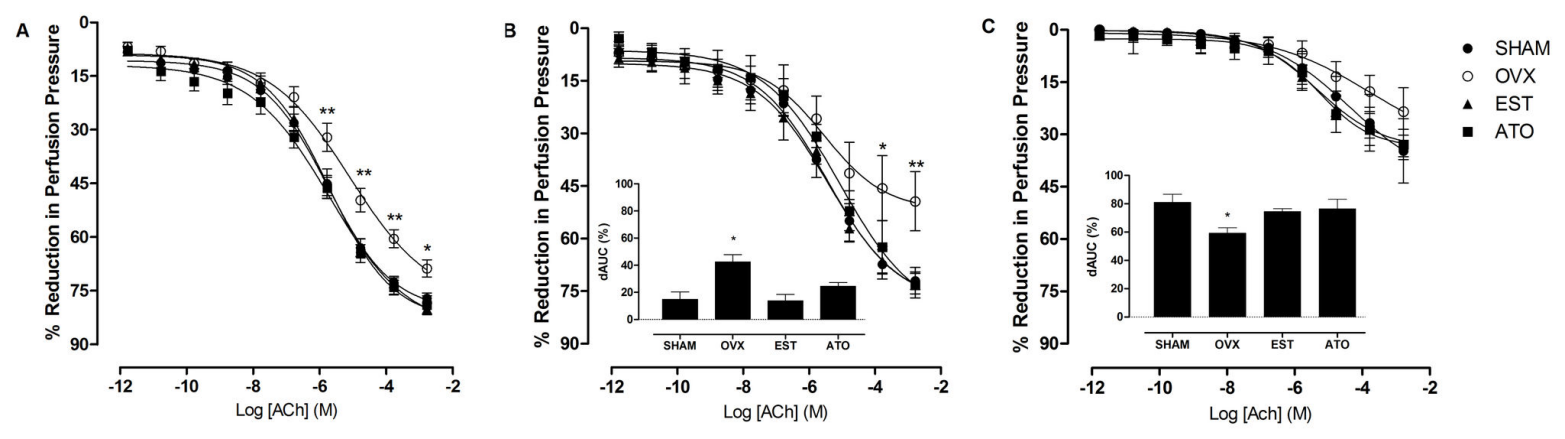
Effect of atorvastatin treatment on endothelium-dependent relaxation caused by acetylcholine in ovariectomized rats. The mesenteric vascular beds (MVBs) from control (SHAM), ovariectomized (OVX) and ovariectomized treated with 17β-estradiol (EST) or atorvastatin (ATO) groups were contracted with noradrenaline (NE) in the absence (A) or presence of aminoguanidine (B) or N^G^-nitro-L-arginine methyl ester (L-NAME) (C). The inset shows differences in the area under the concentration-response curves (dAUC%). The responses are expressed as the percentage of reduction in the perfusion pressure relative to the contractions induced by NE. Each point represents the mean of 6 experiments ± S.E.M. **P<0.01 and *P<0.05 vs. the SHAM group by two-way ANOVA followed by Tukey’s test.

In all experiments using a constant flow rate on a perfused mesenteric vascular bed, we did not observe a change in basal perfusion pressure (mmHg) among the groups (SHAM: 41±1; OVX: 40±1; EST: 40±1; ATO: 39±1; mmHg), neither in the perfusion pressure after the increase of the vascular tone with noradrenaline (SHAM: 144±4; OVX: 143±5; EST: 143±4; ATO: 141±5; mmHg). Acetylcholine induces relaxation in the MVB in a dose-dependent manner (Figure 1 A). In the OVX rats, the endothelium-dependent relaxation after noradrenaline-induced constriction was reduced compared to the SHAM group, while this decreased response was restored in the MVBs from the ATO- or EST-treated ovariectomized rats. There were no changes in pD_2_ values among groups for each drug studied (Table 2). 

**Table 2:** Maximum response (E_max_) and sensitivity (pD_2_) of the concentration-response curves to acetylcholine in the MVBs.

	**Control**		**AG**		**L-NAME**		**L-NAME + INDO**	
	**E_max_**	**pD_2_**	**E_max_**	**pD_2_**	**E_max_**	**pD_2_**	**E_max_**	**pD_2_**
**SHAM**	79.9±3.1	6.01±0.3	76.7±3.8	5.52±0.2	31.6±6.5	5.04±0.3	46.9±8.5	5.55±0.9
**OVX**	65.7±3.4*^#^	5.46±0.2	48.1±5.3**^##^	5.61±0.1	23.8±6.8	4.74±0.3	43.4±6.4	4.94±0.4
**EST**	82.2±2.8	5.59±0.2	77.6±3.3	5.66±0.1	33.9±4.6	5.31±0.3	39.9±6.8	4.93±0.5
**ATO**	87.6±0.3	5.83±0.3	85.0±3.8	5.23±0.1	35.2±5.8	5.23±0.2	35.5±6.4	4.74±0.1

The results are the means ± S.E.M. of 6 experiments. Parameters measured in the control SHAM, ovariectomized (OVX) and ovariectomized treated with 17β-estradiol (EST) or atorvastatin (ATO) groups. E_max_, maximal response (expressed as the percentage of the maximum relaxation induced by acetylcholine); pD_2_, -log one-half E_max_; AG, aminoguanidine; L-NAME, N^G^-nitro-L-arginine methyl ester; INDO, indomethacin. Statistical significance is indicated by **p<0.01 and * p<0.05 *vs.* the SHAM group; ***^##^***p<0.01 and ***^#^***p<0.05 *vs.* the ATO and EST groups (one-way ANOVA followed by Tukey’s test).

### Effect of NOS antagonism on endothelium-dependent relaxation

Nitric oxide modulation of ACh-induced relaxation was evaluated using AG and L-NAME incubation. The MVB preparations treated with these inhibitors showed similar perfusion pressure values (AG: SHAM: 141±4; OVX: 141±7; EST: 140±6; ATO: 143±4; mmHg / L-NAME: SHAM: 143±4; OVX: 152±6; EST: 146±4; ATO: 158±7; mmHg) among the groups. In the OVX rats, ACh relaxation was significantly attenuated by AG, but AG did not modify the response in MVBs from the SHAM group. Treatment with ATO or EST prevented the endothelial alterations observed in the OVX group, as shown by the dAUC values (SHAM: 14.8±5.5; OVX: 42.4±6.8*; EST: 13.8±4.7; ATO: 24.3±2.9; *P<0.05; % dAUC) (Figure 1 B, Table 2). 

L-NAME potentiated the reduction in the vasorelaxation response induced by ACh in all of the groups, but these effects were greater in the OVX group than in the SHAM, ATO, or EST rats, as shown by the dAUC values (Figure 1 C), suggesting a significant influence of NO on the dysfunction observed in the OVX rats. Additionally, neither L-NAME nor AG altered the pD_2_ values after ACh-induced relaxation in the MVBs in any of the groups (Table 2).

### Effect of EDHF and COX inhibition on endothelium-dependent relaxation

**Figure 2:**
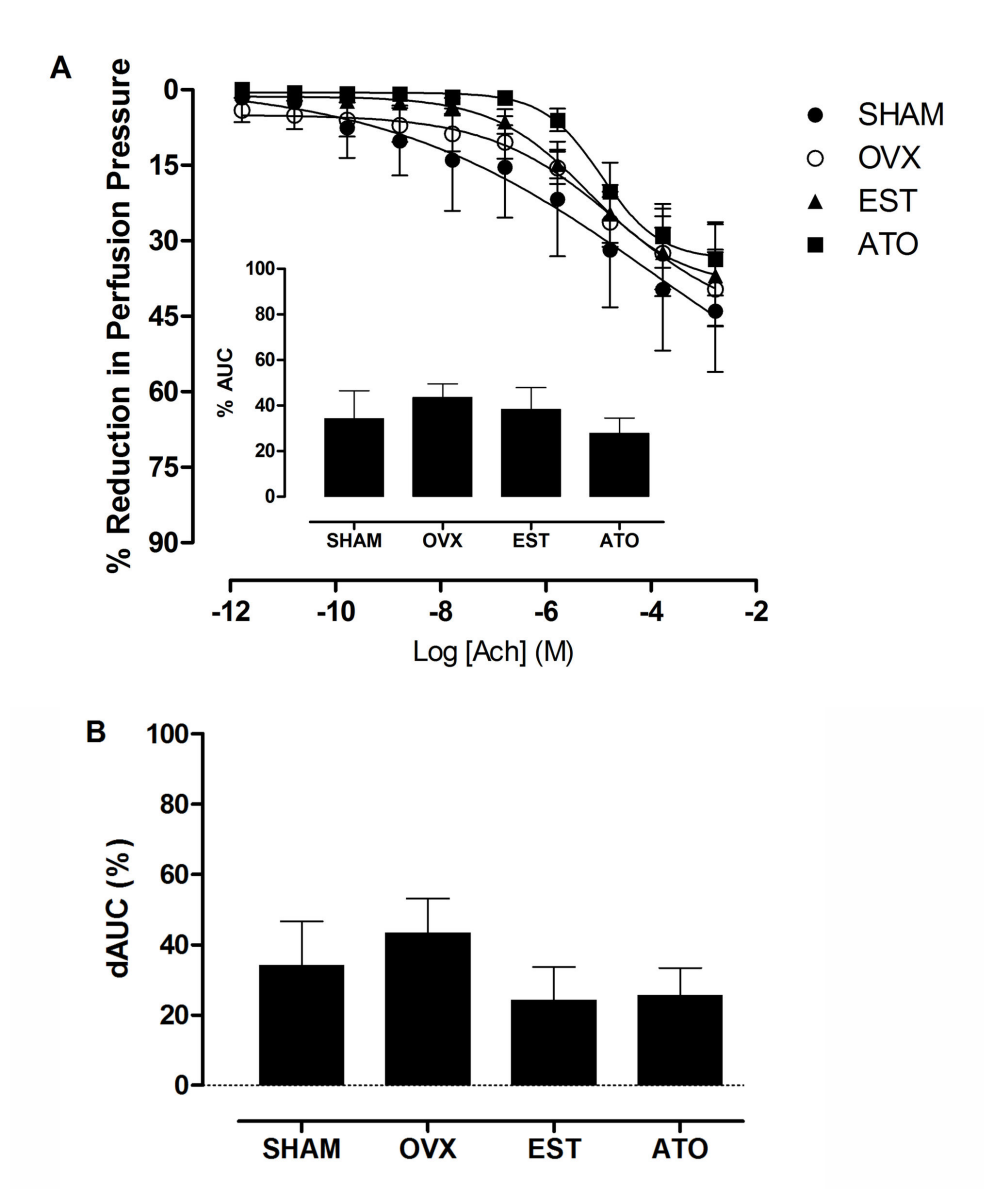
Effect of atorvastatin treatment on EDHF- and prostanoid-mediated relaxation in the MVBs. MVBs from the control (SHAM), ovariectomized (OVX) and ovariectomized treated with 17β-estradiol (EST) or atorvastatin (ATO) groups were contracted with noradrenaline (NE) in presence of N^G^-nitro-L-arginine methyl ester (L-NAME) plus indomethacin. The inset shows the area under the concentration-response curves (AUC%) after this double blockade, which represents the magnitude of EDHF-mediated relaxation (A). The role of prostanoids in MVB relaxation is represented by the difference in the area under the curve (dAUC%) between the groups in the presence of L-NAME and after inhibition with L-NAME plus indomethacin (B). The responses are expressed as the percentage of reduction in the perfusion pressure relative to the contractions induced by NE. Each point represents the mean of 6 experiments ± S.E.M.

The association between L-NAME and INDO was used to investigate the role of EDHF on the decreased response to Ach in the OVX rats. There were not significant differences in the perfusion pressures amongst the four groups after the treatment of the MVB with L-NAME and INDO (SHAM: 147±3; OVX: 138±8; EST: 142±4; ATO: 145±5; mmHg). When applied concomitantly, these drugs failed to further increase ACh-induced relaxation and E_max_ compared to the response to L-NAME in all of the groups (Figure 2 A, Table 2). In addition, the AUC values, which showed the magnitude of EDHF participation in relaxation, were also similar among all of the groups (SHAM: 34.1±12.3; OVX: 43.5±6.0; EST: 38.2±9.6; ATO: 27.8±6.6; %AUC, Figure 2 A, inset), indicating that neither estrogen deficiency nor the treatments affected EDHF. 

The putative role of prostanoids was assessed by the dAUC (%) values in the presence of L-NAME and after inhibition with both L-NAME and INDO, which showed no differences among the groups (Figure 2 B). In addition, no incremental change in E_max_ or pD_2_ of the concentration-response curves occurred in the presence of L-NAME plus INDO compared to L-NAME alone (Table 2). These results indicate the non-expressive participation of prostanoids in the vasodilatory response to acetylcholine in the MVBs from the studied groups.

### Effect of atorvastatin on reactive oxygen species production

**Figure 3:**
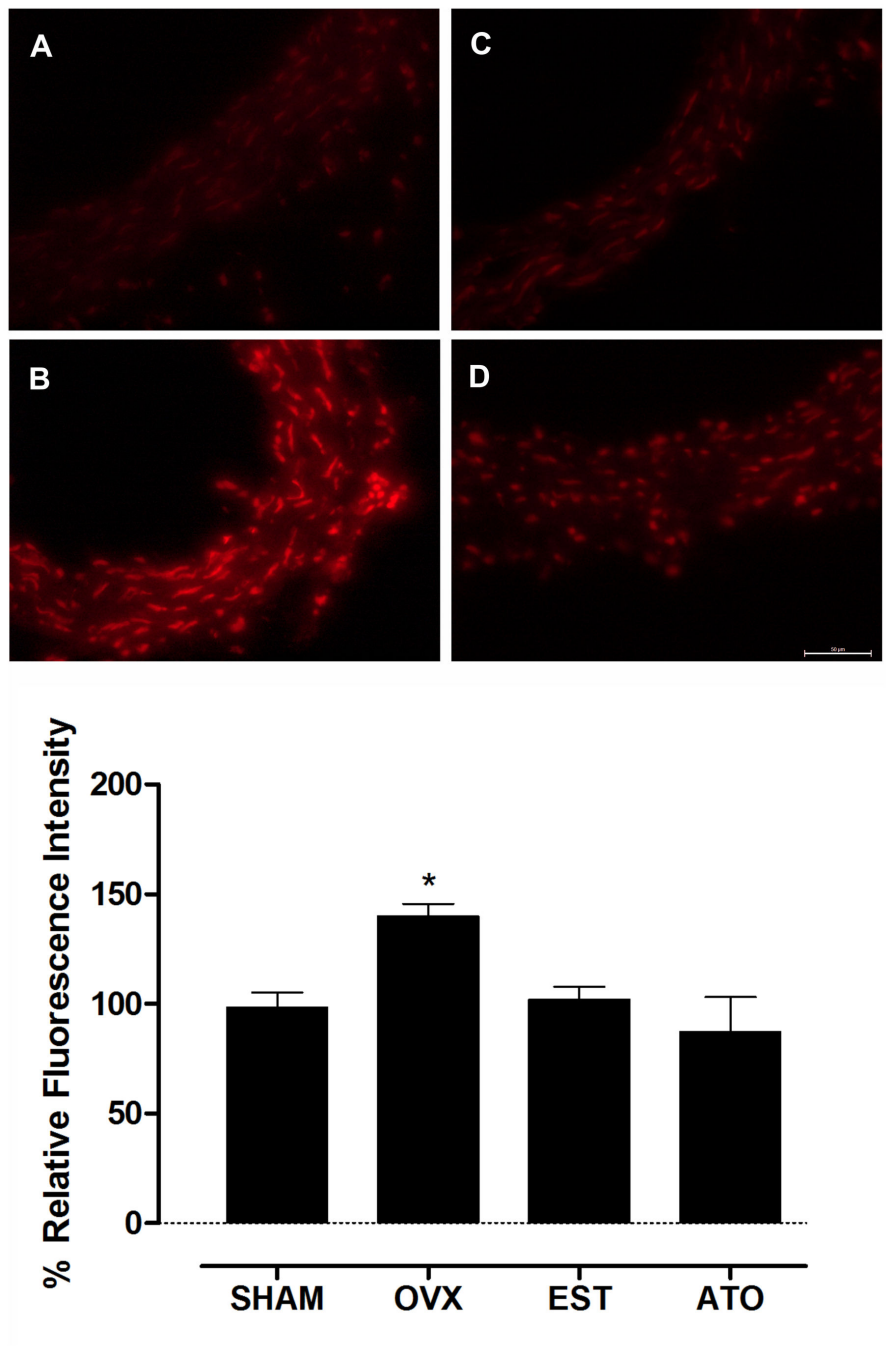
Atorvastatin treatment improves oxidative stress in the mesenteric arteries from ovariectomized rats. Representative DHE staining in mesenteric arteries from the control (SHAM) (A), ovariectomized (OVX) (B) and ovariectomized treated with 17β-estradiol (EST) (C) or atorvastatin (ATO) (D) groups (upper panel). The fluorescent intensity was quantified based on the red signal (magnification x40, lower panel). Each column represents the mean of 6 experiments ± S.E.M., and the results are expressed as the percentage of the SHAM group. *P<0.05 vs. the SHAM group by one-way ANOVA followed by Tukey’s test.

To evaluate tissue ROS production, DHE staining was performed in the mesenteric arteries. At baseline, DHE red fluorescence analysis revealed an increased production of superoxide anion from the mesenteric vessels in the OVX rats compared to the SHAM rats. Treatment with ATO or EST for 2 weeks corrected the enhanced ROS production in the mesenteric arteries from the OVX rats (Figure 3).

### Western blot analysis of eNOS, iNOS, COX-2 and NADPH oxidase

**Figure 4:**
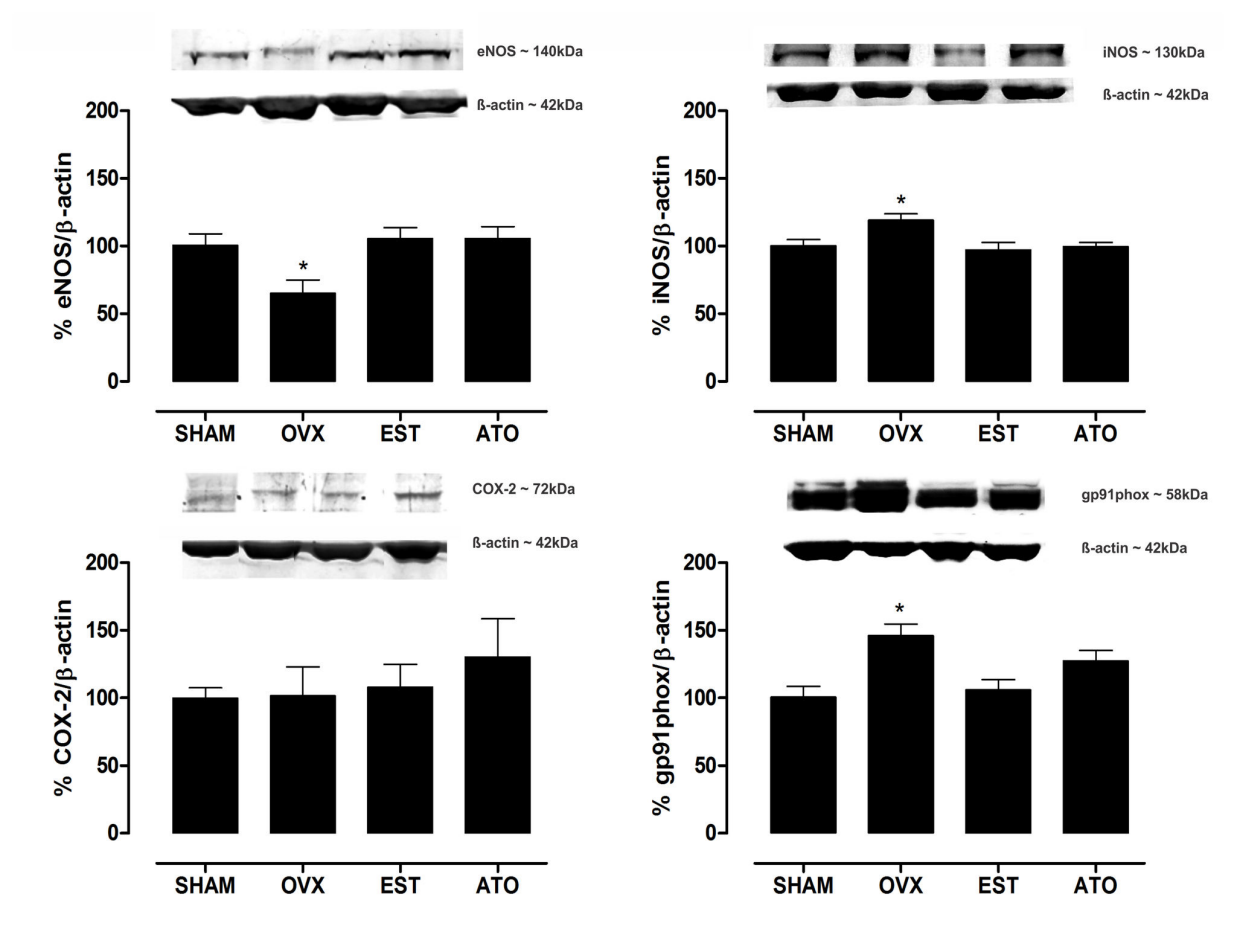
Effect of atorvastatin treatment on the expression of signaling pathway proteins. Western blot analysis of eNOS, iNOS, COX-2 and NADPH oxidase (gp91phox) in the mesenteric vascular beds from the control (SHAM), ovariectomized (OVX) and ovariectomized treated with 17β-estradiol (EST) or atorvastatin (ATO) groups (upper panels, representative blots). The column graphs refer to the densitometric analysis of the bands normalized to total β-actin expression. Each column represents the mean of 6 experiments ± S.E.M., and the results are expressed as the percentage of the SHAM group. **P<0.01 and *P<0.05 vs. the SHAM group by one-way ANOVA followed by Tukey’s test.

Ovariectomy reduced eNOS and increased iNOS protein expression in the mesenteric branches (Figure 4); in addition, gp91phox protein expression, a subunit of the NADPH oxidase complex, was increased in the OVX group. The ovariectomized rats treated with ATO or EST showed similar protein expression as the SHAM animals. There was no change in COX-2 protein expression among the groups (Figure 4).

## Discussion

The present study indicates that the treatment with atorvastatin is sufficient to reverse the endothelial dysfunction observed in female rats with estrogen deficiency. Importantly, our experiments also demonstrated that functional changes in the MVBs were associated with molecular adaptations. Namely, our major novel finding is demonstrating that increased ROS production, NADPH oxidase and iNOS overexpression and reduced eNOS expression in OVX mesenteric vessels, which can lead to reduced NO availability, were restored by atorvastatin and estrogen replacement.

The decrease in the relaxation response after acetylcholine administration to MVBs from ovariectomized rats is most likely due to increased negative endothelial modulation [9,20,43,45], which is highlighted by the increase in oxidative stress and reduction in endothelial relaxation factors observed in estrogen deficiency. In fact, previous reports have suggested that ovariectomy might contribute to impaired endothelial function by reducing EDHF [6,8,46] and NO [47,48] and increasing vasoconstricting prostanoids [7,49] and ROS [9,50,51] in different conductance or resistance vessels. 

There is particular interest in the endothelial NO system and endogenous estrogen deprivation, in light of the relevant functional role of endothelial NO on regulating vascular tone [7,52]. However, most studies have focused on analyzing a single aspect of the system and have not examined an entire vascular bed, as in our study. Thus, estrogenic derivatives have been reported to increase [53–55] or not affect [56,57] NO vascular modulation. Regarding our results from the MVBs, we reported that, similar to estrogen treatment, atorvastatin was able to normalize endothelial function and restore NO availability in the OVX rats, as documented by aminoguanidine and L-NAME inhibition of iNOS and NOS ACh-induced relaxation, respectively. In addition, we observed that the magnitude of the effect of L-NAME (%dAUC) was lower in the OVX rats, suggest that reduced NO bioavailability is possibly the major determinant of endothelial vascular alterations in the OVX rats. 

The expression of iNOS has been documented in vascular endothelial and smooth muscle cells after either inflammatory or cytokine stimulation [58–60], in female rats with estrogen deficiency state [8,61]. Our study indicates a higher putative participation of NO from iNOS in the relaxation responses to ACh in the OVXs. However, the contribution of iNOS was not able to compensate the NO- deficiency observed in the OVXs once they presented reduced relaxation responses to ACh.

In conjunction with our functional data, the DHE analysis revealed that the enhanced production of ROS in the OVX rats was dramatically reduced by atorvastatin. Consistent with these results, we found that statin treatment reduced NADPH oxidase and iNOS expression in the mesenteric vessels. Moreover, ATO-treated OVX rats had normalization of eNOS expression, similar to the estrogen-treated rats. Taken together, these results showed that atorvastatin was able to reverse endothelial dysfunction in resistance vessels from OVX rats by restoring NO availability, preventing oxidative stress and normalizing the expression of important signaling pathways enzymes.

The present study agree with and extend previous evidence supporting statins’ effects on modulating oxidative stress [18,26,27,31,62]. Previous studies have demonstrated that statins enhance eNOS phosphorylation to increase the level of activated eNOS in aortic rings from male SHR [63], increase NO in VSMCs [14], and might inhibit iNOS expression and induction in blood vessels [14,64,65], independent of this drug’s effect on cholesterol. Moreover, statins have been reported to be able to inhibit the activation and translocation of Rac 1 from the cytosol to the cell membrane, which is critically involved in the activation of the NADPH complex [26,31,66]. Furthermore, others studies have indicated that the antioxidant effects of these drugs extend beyond reduced NADPH oxidase activity in VSMCs and affect Nox1 and p22phox [14]. Statins have also been shown to act on radical scavenging enzymes, enhancing Cu/Zn-SOD and EC-SOD expression in the mesenteric arteries of Ang II-treated rats [22] and increase HO-1 and catalase activities in human osteoblastic cells [67].

Several reports have also suggested that ROS production during estrogen deprivation may lead to the development of endothelial dysfunction [68–70], especially those ROS derived from NADPH oxidase, which is the main source of superoxide anion in the vascular system [71]. Our findings indicate that the ability of atorvastatin in restoring the expression of NADPH oxidase, similar to estrogen, is the most likely mechanism by which this drug was able to reduce the vascular oxidative stress in the OVX rats. Our results are consistent with those obtained in other studies, which demonstrated the potential antioxidant effect of atorvastatin in vascular segments, such as the aorta and mesenteric arteries from male normotensive and hypertensive rats [22,26,27]. However, statins seem to have an antioxidant effect not just in the cardiovascular system because statins have been shown to reduce oxidative stress in plasma from male Wistar rats [22] and to attenuate ROS-induced osteoporosis in ovariectomized female Sprague-Dawley rats that received simvastatin for 8 weeks [67]. 

Despite these ﬁndings, the involvement of other vasoactive substances that counteract the vasodilatory effect of ACh cannot be ruled out. Our study examines the impact of atorvastatin on endothelial dysfunction in ovariectomized rats and its effect on the NO pathway and intravascular oxidative stress, without modulating EDHF and prostanoid function and COX-2 vascular expression. The inhibitory effect of L-NAME on ACh-induced relaxation was not modified by COX blockade, based on pharmacological analysis using indomethacin. The double blockade (L-NAME plus INDO) revealed that EDHF was not altered by estrogen deficiency or atorvastatin treatment in the MVBs, as also shown by the %dAUC values, suggesting that NO might be reduced in the OVX rats independent of EDHF, which has been reported by others [72]. Furthermore, we demonstrated that vasodilatory prostanoids, such as prostacyclin, play a negligible role in the agonist-induced, endothelium-dependent relaxation in female rat MVBs regardless of the estrogen status, according to a study by Liu et al. [73]. In addition, atorvastatin has been reported not to influence the prostanoid pathway (COX-2 expression) in small mesenteric arteries from normotensive male rats [27].

In conclusion, our results indicate that endothelial dysfunction secondary to estrogen deficiency in ovariectomized rats was normalized by atorvastatin through NO-mediated mechanisms and reduced oxidative stress. This experimental evidence partially explains the atheroprotective properties of statins independent of their effects on cholesterol and provides insight into the development of new therapeutic strategies to treat menopausal endothelial dysfunction. Large clinical trials are necessary to confirm if these beneficial effects are maintained in a long period.
